# Early age decline in DNA repair capacity in the liver: in depth profile of differential gene expression

**DOI:** 10.18632/aging.101120

**Published:** 2016-11-30

**Authors:** Avital Guedj, Anat Geiger-Maor, Hadar Benyamini, Yaval Nevo, Sharona Elgavish, Eithan Galun, Hagai Amsalem, Jacob Rachmilewitz

**Affiliations:** ^1^ Goldyne Savad Institute of Gene Therapy, Hadassah-Hebrew University Medical Center, Jerusalem, Israel; ^2^ Bioinformatics Unit, of the I-CORE Computation Center, the Hebrew University and Hadassah Hebrew University Medical Center, Jerusalem, Israel; ^3^ Department of Obstetrics and Gynecology, Hadassah University Hospital-Mount Scopus, Jerusalem, Israel

**Keywords:** DNA repair, aging, γH2AX, KAP-1, p53, RNAseq

## Abstract

Aging is associated with progressive decline in cell function and with increased damage to macromolecular components. DNA damage, in the form of double-strand breaks (DSBs), increases with age and in turn, contributes to the aging process and age-related diseases. DNA strand breaks triggers a set of highly orchestrated signaling events known as the DNA damage response (DDR), which coordinates DNA repair. However, whether the accumulation of DNA damage with age is a result of decreased repair capacity, remains to be determined. In our study we showed that with age there is a decline in the resolution of foci containing γH2AX and pKAP-1 in diethylnitrosamine (DEN)-treated mouse livers, already evident at a remarkably early age of 6-months. Considerable age-dependent differences in global gene expression profiles in mice livers after exposure to DEN, further affirmed these age related differences in the response to DNA damage. Functional analysis identified p53 as the most overrepresented pathway that is specifically enhanced and prolonged in 6-month-old mice. Collectively, our results demonstrated an early decline in DNA damage repair that precedes ‘old age’, suggesting this may be a driving force contributing to the aging process rather than a phenotypic consequence of old age.

## INTRODUCTION

Aging has been defined as a progressive decline in function at the cellular, tissue, and organism level as well as a loss of homeostasis. Aging at the molecular level is characterized by the gradual accumulation of molecular damage caused by environmental and metabolically generated free radicals [1]. While all biological macromolecules are susceptible to corrup-tion, damage to a cell's genomic DNA is particularly harmful. Age-related accumulation of unrepaired DNA breaks that lead to increased frequency of mutations and genomic instability [2–5], has long been proposed as a major source of stochastic changes that can influence aging (reviewed in [6, 7]).

DNA damage appears to be a central factor of aging, acting as both the cause and the consequence of aging. On the one hand, aging is a life-long process, influenced continually by environmental conditions. Factors such as diet, lifestyle, exposure to radiation and genotoxic chemicals seem to have a significant influence on the accumulation of DNA damage noted with age [8]. In turn, age-related accumulation of DNA damage may cause progressive and irreversible physiological attrition and loss of homeostasis, hence accelerating the aging process [9]. In this regard, it is important to note that most human premature aging diseases are associated with defects in the DNA damage repair mechanism [10–12]. Likewise, mice with genetic deficiencies in DSBs repair have much shorter lifespans than the wild-type [13]. Mice deficient in the DNA excision-repair gene Ercc1 have a median life span of 5-6 months [14]. Moreover, a recent study has provided direct evidence for the role of DSBs, the most dangerous type to the cell, in aging. The authors demonstrated that shortly after the induction of DSBs (as early as 1 month) livers of 3-month-old mice developed many phenotype characteristics of liver aging, indicating that DSBs alone can drive the aging process [15].

Hence, DNA damage is likely a key contributor to the aging process, however, it still remains to be determined what causes DNA damage accumulation with age and specifically whether compromised DNA repair leads to persistent DNA damage. A number of studies provided evidence supporting the notion that DNA damage repair activity declines in both aged mice and humans. These studies showed that diminished rates of DNA repair in aged animals results from reduced efficiency and fidelity of the molecular machinery that catalyzes DNA repair [16–19]. Further studies suggested that important proteins participating in various DNA repair processes exhibit an age-related decline in both basal and damage-induced expression levels [20, 21]. Other studies suggested an impaired or delayed recruitment of DNA repair factors, such as RAD51, to the DNA damage sites [20–23]. Regardless of the underlying mechanism, these studies demonstrate that old age is associated with decreased DNA damage repair capacity.

In a recent study, we demonstrated in 1-month-old mice that diethylnitrosamine (DEN)-induced DNA damage is resolved within 6 days, reflecting the efficiency of the DNA-repair mechanisms [24]. In the present study we extended this earlier observation to mice of various ages, in order to determine how age affects the extent of DSBs generation and the kinetics of the resolution. Rather than looking at the decline in DNA damage repair activity in ‘old’ mice we specifically looked for changes that occur in an age-dependent manner. Using this approach we demonstrated a surprisingly early-age decline in DNA damage repair and alteration in transcriptional profiles that precedes old age.

## RESULTS

### Age-dependent decline in DNA damage repair

To test the efficiency and kinetics of DNA damage repair *in vivo*, we induced DNA damage in mouse livers by a single injection of DEN. We performed immuno-fluorescence staining to detect cells containing phosphorylated histone H2AX (γH2AX) in liver tissue sections at various time points following the DEN injection. Using this mouse model we have previously demonstrated that cells positive for γH2AX appear 24h after DEN injection and persist up to three days post-injection. The DNA damage was resolved by day six post DEN treatment, and at this point in time the number of cells harboring γH2AX foci and their relative intensity significantly declined, leaving only a few cells with detectable foci [25]. One month-old mice were used in these previous experiments. In order to test whether DNA damage response declines with age, we compared the efficiency of DNA damage repair following DEN injection in 1-, 3-, 6- and 12-month-old mice.

**Figure 1:**
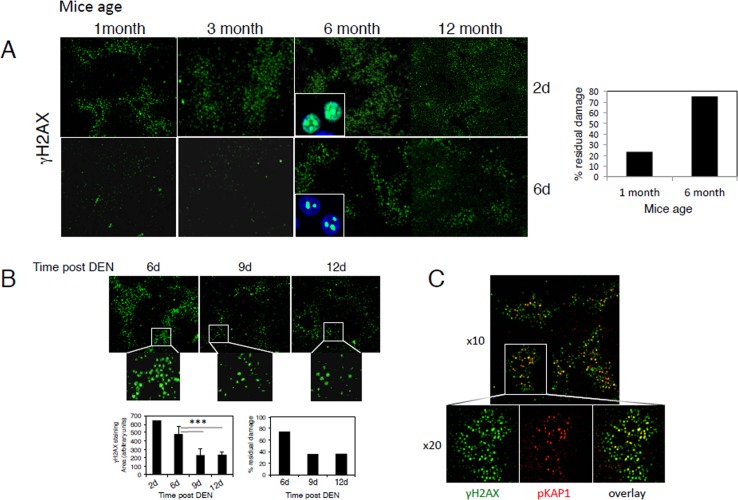
Age-dependent decline in the repair of DEN-induced DNA damage (**A**) Mice at the indicated ages were injected with DEN and sacrificed after two and six days. Representative low power filed images of γH2AX staining in liver sections are shown (x4 objective). The percentage of DNA damage repair was calculated by dividing the amount of DNA damage at the peak of damage after 48h and the residual damage after the resolution phase 6 days after DEN treatment. The percentage of residual damage in 1-month compared to 6-month-old mice is shown (right panel). Insets (high power images -objective x40) depict γH2AX (green) in hepatocyte nuclei (DAPI; blue). (**B**) Six-month-old mice were injected with DEN and the levels of residual damage after 6, 9 and 12 days were determined as above. Insets depict the lower density and intensity of γH2AX staining in 9 and 12 days compared to 6 days post DEN. Graph (lower left panels) shows γH2AX staining areas 2-12 days after DEN injections in 6-month-old mice (average ± STD). The percentage of DNA damage repair was calculated as in A (lower right panel). An average of at least four mice in each group is shown. *** p<0.0005. (**C**) Representative image of γH2AX (green) and pKAP-1 (red) staining demonstrating pKAP-1 foci overlapping with γH2AX at sites of DSBs.

Since DEN itself does not exert hepatocyte toxicity, it needs to be metabolically activated by cytochrome P450 enzymes (CYP) in the liver, predominantly by the cytochrome P450 2E1 (CYP2E1), resulting in DNA-adducts formed through an alkylation mechanism [26]. We, therefore, analyzed expression of this cytochrome P450 isoform by quantitative real time PCR. We did not detect any significant alterations in expression levels of CYP2E1 between mice at various ages (Supplementary Figure S1A). Furthermore, out of 117 CYP isoforms that were detected in an RNAseq analysis only 15 were differentially expressed between 1 month and 6 month-old mouse livers, and only 4 were upregulated with age. Consistent with similar metabolic activation of DEN at all ages, we detected comparable levels of DNA double strand breaks, as indicated by γH2AX immuno-fluorescence, 48h after DEN insult. There was no significant difference in the extent of initial damage noted in the liver two days after DEN injection in mice of all ages, however, the extent of residual DSBs six days after DEN injection was significantly higher in 6- and 12-month old mice as compared to younger mice (Fig. 1A). The extent of DNA damage repair was calculated by dividing the area of DNA damage at the peak of damage (after 48h) and the residual damage after the resolution phase (6 days post DEN treatment). While, approximately 80% of the initial damage was resolved in 1- and 3-month-old mice by day 6, only ~25% of the damage was resolved in 6- and 12 month old-mice although the intensity of γH2AX staining was somewhat lower compared to 48h post DEN treatment (Fig. 1A).

Interestingly, the level of residual damage in 6-month-old mice further declined to around 40% at days 9 and 12 after DEN treatment and the intensity of residual foci were further reduced as compared to day 6 (Fig. 1B), suggesting that with age DNA damage repair process is both impaired and significantly delayed.

The heterochromatin protein KRAB-associated protein (KAP-1), a co-repressor of gene transcription, is phosphorylated by Ataxia telangiectasia mutated (ATM) at serine 824 in response to DNA damage. Phosphorylated KAP-1 (pKAP-1) forms foci overlapping with γH2AX at sites of DSBs [27, 28] and was suggested to control DNA repair in hetero-chromatin [29, 30]. We, therefore, performed immuno-fluorescence staining to detect pKAP-1 in liver tissue sections from various ages and at various time points following DEN injection. Like γH2AX, pKAP-1 foci were clearly evident after DEN injection, and were co-localized with relatively intense γH2AX-positive nuclei (Fig. 1C). The time-dependent appearance and recovery of pKAP-1 followed the same pattern and kinetics as γH2AX at all mouse ages (Supplementary Fig. S1B and not shown), thus confirming γH2AX results. However, pKAP-1 foci seemed to resolve better than γH2AX foci that are more persistent (Supplementary Fig. S1B).

### DEN induces age-dependent changes in gene expression profiles

**Figure 2:**
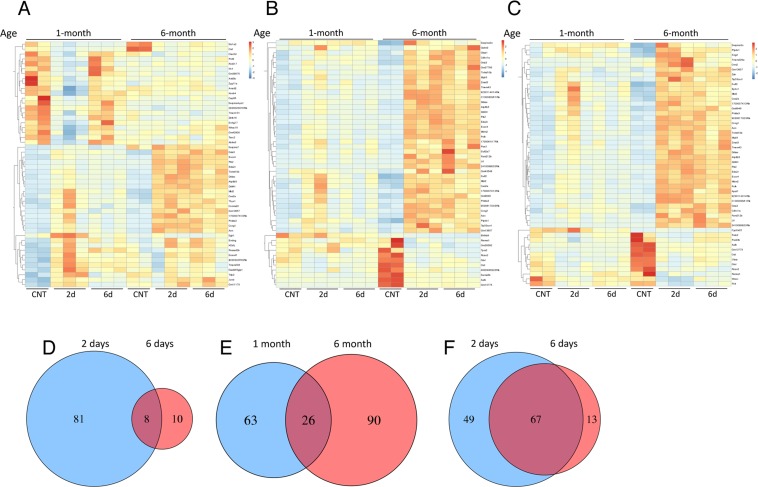
DEN-induced, age-dependent differential gene expression profiles Heat maps depicting RNAseq gene expression profiles of mouse liver after DEN treatment. Hierarchical clustering analysis of top 50 differentially expressed transcripts in mouse livers 2 days after DEN treatment compared to control untreated livers in 1-month-old mice (**A**), 6-month-old mice (**B**) or genes differentially expressed at day 6 after DEN treatment compared to control in 6-month-old mice (**C**). Venn diagrams show the number of genes detected as differentially expressed by the DESeq2 package. (**D**) Comparing day 2 to day 6 in 1-month-old mice. (**E**) Comparing day 2 in 1-month and 6-month-old mice. (**F**) Comparing day 2 to day 6 in 6-month-old mice; demonstrating resolution of the response in day 6 in 1-month but not 6-month old mice with relative little overlap in the transcriptional response between the two ages at day 2.

To more sensitively detect potential age-related changes in DEN-induced DNA damage response in the liver we used RNAseq analysis. We specifically looked for genes that are differentially expressed two and six days after DEN treatment in 1- and 6-month-old mice (the earliest age where DNA damage resolution decline was detected). Two days after DEN-treatment 89 differentially expressed genes were identified in livers of 1 month old mice that were classified into two clusters based on the expression profile using hierarchal clustering. One cluster contained 47 genes that were downregulated as compared to the control and the second cluster contained 42 upregulated genes (Fig. 2A). Overall, most differentially expressed genes (both up- and down-regulated) returned to baseline levels by day 6, thus demonstrating that the response to DEN was largely resolved at the transcriptome level. This hypothesis was reinforced by the finding that only eight of the 89 differentially expressed genes at day 2 were also differentially expressed at day 6, and all together only 18 genes were found to be differentially expressed in day 6 as compared to the control (Fig 2D). Remarkably, most downregulated genes in 1-month-old mice were not differentially expressed in 6-month old mice. In contrast, most upregulated genes in 1 month old mice were also upregulated in 6-month old mice, however they did not return to basal levels at day 6 (Fig. 2A).

In 6-month-old mouse livers, 116 genes were significantly differentially expressed at day 2 in contrast to 1-month-old mice where the majority of these genes (both up- and down-regulated) did not resume basal expression levels at day 6 (Fig. 2B). Surprisingly, despite the comparable response to DEN at day 2 (Figure 1A), 1 month and 6 month old mice shared only 26 differentially expressed genes at day 2 (Fig. 2E). Comparing 6 days post DEN to the control revealed 80 differentially expressed genes, 67 of which were shared with the differentially expressed genes at day 2 (Fig. 2F). Surprisingly, the fold change in the expression levels of these shared genes did not significantly reduce at day 6 as compared with day 2 (Supplementary Fig. S2A), demonstrating that between day 2 and day 6 the response did not significantly decline.

In keeping with the observed age-related decline in DNA damage repair, the data demonstrated a marked difference in gene expression pattern in response to genotoxic damage between 1 and 6 month old mice and the extent to which the response is resolved.

To validate RNAseq results, we confirmed the transcriptional levels of genes representing different expression patterns by qRT-PCR (Supplementary Fig. S2B). These genes exhibited differential expression in qRT-PCR that was consistent with the RNAseq data, indicating good concordance of both methods. In addition, we tested the pattern of expression of these genes in 3- and 12-month old mice and demonstrated that they followed similar pattern of expression as 1- and 6-month-old mice, respectively, thus closely paralleling their DNA damage resolution kinetics. Moreover, for genes that did not resume basal levels in 6-month-old mice by day 6 we further analyzed their expression at day 9 and 12. In accordance with the reduced levels of DSBs in the liver at these time points (Fig. 1B), the level of expression was also slightly reduced at day 12 (but did not return to basal levels).

### Functional analysis of differentially expressed genes

**Figure 3:**
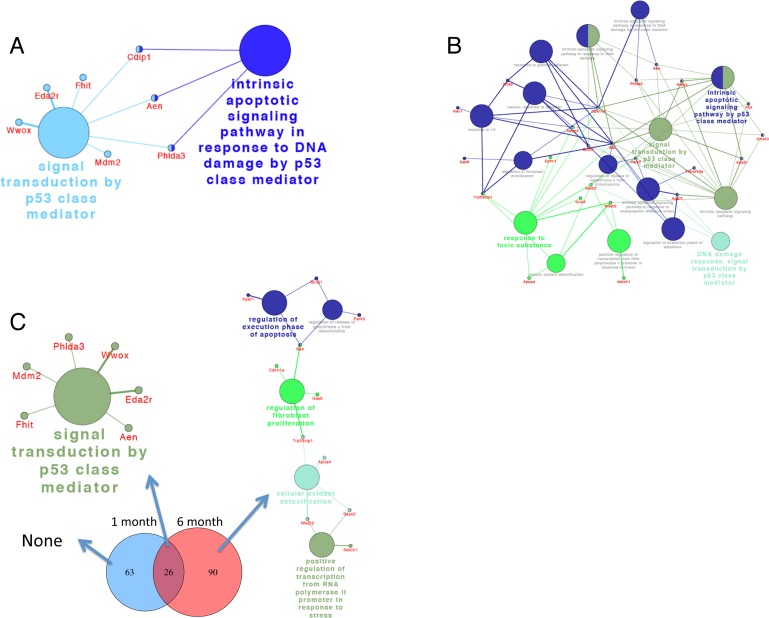
ClueGo network analysis of differentially expressed genes reveal robust activation of p53-related pathways Differentially expressed genes 2 days after DEN in 1-month old (**A**) and 6-month-old mice (**B**) and common and exclusively differentially expressed genes between the two ages (**C**), were annotated in the context of the GO database, and the relationships among these annotated terms were calculated and grouped by ClueGO to create an annotation module network. Functionally grouped networks with pathways and genes are shown. The node size represents the term enrichment significance.

After identifying the profiles of differentially expressed genes for each comparison, we performed functional enrichment analysis to reveal transcripts putatively involved in potential relevant biological processes, signaling pathways and networks using ClueGO in Cytoscape. The Venn diagram analysis shown in the previous section allowed us to uncover common and exclusively differentially expressed genes between the two mouse ages, underscoring potential common and exclusive biological functions regulated in each case. Genes associated with p53 signaling pathway and intrinsic apoptotic signaling pathway in response to DNA damage by p53 were significantly enriched 2 days after DEN injection in both 1-month and 6-month old mice (Fig. 3A-B). However, in 6-month old mice a more robust response was observed. In addition to these two pathways other related pathways and biological functions were also activated including the response to toxic substance, response to UV and gamma radiations and others (Fig. 3B). Consequently, the 26 common genes between the two groups were also associated with signal transduction by p53 (Fig. 3C). The fold change in expression of these shared genes was significantly higher in 6-month-old mice as compared to 1-month-old mice (Supplementary Fig. S3). No significant enriched signaling pathway or biological function was identified among genes that are unique to 1-month-old mice, whereas genes unique to 6-month old mice were enriched for regulation of execution of apoptosis, regulation of fibroblast proliferation, cellular oxidant detoxification, and positive regulation of transcription from polymerase II promoter in response to stress (Fig. 3C). Although the number of common genes between the two ages is surprisingly low, they share a similar functional response based on p53 module with an overall more robust response in 6-month old mice.

**Figure 4:**
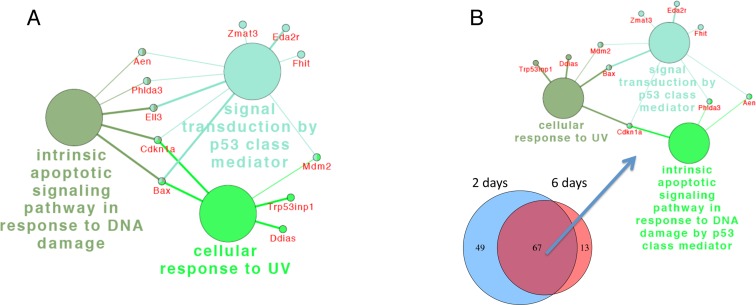
ClueGo network analysis of differentially expressed genes reveal sustained activation of p53-related pathways Annotation module network generated as in Fig. 4 for differentially expressed genes 6 days after DEN (**A**) and for the 67 common transcripts for 2 and 6 days after DEN in 6-month-old mice (**B**), demonstrating that in contrast to 1-month-old mice p53-related pathways did not resolve in 6-month-old mice. The node size represents the term enrichment significance.

At day 6 after DEN treatment no significantly enriched pathway was detected in 1-month old mice. The most relevant biological processes found in genes differentially expressed in 6 month-old mice 6 days after DEN were p53-signaling pathway, intrinsic apoptotic signaling pathway in response to DNA damage by p53 and cellular response to UV (Fig. 4A). These pathways were shared between the two time points and were enriched in differentially expressed genes common to day 2 and 6 (Fig. 4B).

Ingenuity® Pathway Analysis (IPA), pathway and upstream regulator analysis of differentially expressed genes, further corroborated these findings demons-trating a more robust response in 6-month-old mice that was not resolved by day 6 after DEN treatment. In general, most upstream regulators identified by this analysis were associated with p53 signaling pathway and agents inducing DNA damage (Supplementary Fig. S4A-C).

Additionally, we combined differentially expressed genes from all 4 groups and analyzed putative protein interaction network analysis. This analysis once again confirmed the results and highlighted a common interaction network that related to the genes involved with p53 signaling and intrinsic apoptotic pathways (Supplementary Fig. S4D).

Collectively, the transcriptional data correlates well with the relative inefficient resolution of DSBs in 6 month-old mice. Surprisingly, despite the reduced DNA damage response in this age it is accompanied with a robust early (2 days after DEN) and prolonged p53 signaling response.

## DISCUSSION

There is mounting evidence for an age-dependent accumulation of DNA damage and somatic mutations that in turn can drive and exacerbate cellular and organism aging. However, it is still unclear whether this accumulation is the result of an inherent imperfection of DNA repair and therefore the decline in DNA damage repair contributes to organism aging or is it a consequence of old age. Previous studies in aged mice and humans demonstrated a decrease in the capacity to process damaged DNA in part due to reduced expression of various critical DNA repair proteins [20, 21].

While previous studies compared two age groups, young (2-4 months) animals versus aged ones (typically around 20-28 months), we chose to investigate age as a variable and not necessarily address aging in the context of ‘old age’. We, therefore, evaluated the effect of age on DNA repair activity by exposing mice of various ages, ranging from one to 12 months of age, to the carcinogen DEN. Collectively, our data revealed a decline in DNA damage repair capacity starting at a surprisingly early age of 6-months (obviously not considered ‘old’).

The question is whether the elevated DNA damage resolution seen in 1 month-old mice is a consequence of continued developmental changes that would stabilize with maturity, and therefore not related to differences in age *per-se*. To rule out this possibility, we also tested 3 month-old mice representing fully mature, young mice and demonstrated a similar DNA damage repair capacity and differential gene expression pattern as 1-month-old mice.

A potential concern with the use of DEN relates to its activation by members of the CYP enzymes and specifically by CYP2E1 and the possibility that age-related differences in enzyme expression and activity may occur. While we tested the levels of expression of members of this family in the liver and demonstrated that transcript levels of most CYPs and specifically of CYP2E1, the major DEN-activating enzyme, remains constant with age, it is possible that differences in enzyme activity do exist. Furthermore, we cannot completely rule out the possibility that other age-related factors may affect the generation of DEN-induced DNA breaks. Despite these concerns, our findings demonstrated that initial levels of DSBs in response to DEN are similar in mice of all ages and at both 25mg/kg and 10mg/kg DEN (not shown), as evident from γH2AX and pKAP-1 staining. Hence, we suggest that in our experiments mice at various ages exhibit similar sensitivity to this DNA-damaging agent.

Despite these apparent similar levels of initial DNA damage between the various ages 2 days after DEN injection, gene expression in response to DEN varied significantly and only 26 genes that represent 14.5% of the differentially expressed genes are shared between 1-month and 6-month old mice. Importantly, only 25% of the genes that are differentially expressed in 1 or 6-month-old mice 2 days after DEN were also differen-tially expressed in an age-dependent manner in control naïve livers, suggesting that differences in the response to DEN cannot entirely be attributed to basal age-related changes. Notably, in accordance with their respective DNA damage resolution data, as per γH2AX and pKAP-1 immunofluorescence staining, almost all the genes that were differentially expressed in response to a challenge with DEN resumed basal levels by day 6 in 1-month old but not in 6-month-old mice. Hence, confirming the reduced damage resolution capacity observed at 6-months of age.

Interestingly, as mentioned above, mice deficient in Ercc1 died at the age of 5-6 months [14], surprisingly resembling the age in which the decline in DNA damage repair is observed. Moreover, a recent study reported that a low caloric diet tripled the median lifespan of these mice and significantly reduced the number of γH2AX foci and various other aging associated characteristics [31]. These findings suggest a possible role for cell non-autonomous mechanisms that are responsible for the age-related decline in DNA damage (possibly taking place by the age of 6 months) in addition to the cell-intrinsic decrease in the expression and function of proteins participating in the DNA repair process (such as Ercc1).

Among the DNA lesions, DSBs are the most toxic forms of DNA damage, presenting a serious threat to genome stability and cell viability. Consequently, efficient, accurate and timely processing and repair of DSBs is essential for maintaining genomic stability and cellular fitness. DSB repair usually occurs in two phases; the first operates with high fidelity that promotes rapid and efficient rejoining and the late error-prone phase takes place when rejoining is delayed [32]. An interesting feature of DNA resolution revealed in our study is the significant delay in the resolution of DSBs from 6 days in 1- and 3-month old mice to around 12 days in 6 and 12-month old mice. In support of this finding, a previous study found age-dependent differences already in the early steps of γH2AX foci formation. The authors demonstrated a delay in the recruitment of DSB repair proteins and slower growth of γH2AX foci in older donors [23], suggesting an age-dependent decrease in the efficiency of this process that may contribute to genome instability.

In the past few years, the complex interplay between DNA damage, DNA-repair mechanisms and cellular fate has become evident. A key factor in the network responding to DNA damage is the tumor suppressor p53 that dynamically responds to DSBs [33–35]. The specific dynamics of p53 were found to depend on the extent and persistence of damage and leads to the expression of a different set of downstream genes [36, 37], that in turn activates alternative cellular outcomes ranging from DNA repair, transient cell cycle arrest, or in the case of unresolved damage senescence and apoptosis [38, 39].

Several reports suggested a role for p53 in the reduced ability to process DNA damage in aged animals based on the observation that declined damage repair is associated with decreased constitutive mRNA and protein levels of p53 as well as reduced accumulation of p53 in response to DNA damage [20, 21]. Hence, these data suggested that with age there is less induction of p53 following DNA damage.

In contrast, gene expression data presented in this study revealed that the induction of p53-signaling pathway is more pronounced in 6-month old mice- as compared to 1-month-old mice exposed to DEN. Moreover, we demonstrated sustained p53-pathway activity in 6-month but not in 1-month-old mice 6 days after DEN injection. This robust and prolonged p53 activity in the older mice may reflect their inability to efficiently repair damaged DNA and the continuous presence of unrepairable DSBs.

While reduced DNA damage repair and the accumulation of DNA damage has been associated with aging it is not clear whether decreased DNA repair is itself a symptom or a cause of aging. Reduced DNA damage repair and specifically the highly toxic DSBs will most likely result in an increased number of senescent cells and accumulation of somatic mutations that can give rise to functional impairment of tissues that drive aging. The finding that the decline in DNA-repair capacity takes place early in life and precedes ‘old age’ raises the possibility that the decreasing efficiency of this process may be a contributing and accelerating factor that causes degeneration of cells and tissues associated with aging and age related diseases.

## METHODS

### Animal model

Experimental protocol was approved by the Hebrew University Institutional Animal Care and Ethical Committee. For the induction of DNA damage *in vivo*, 1,3,6 and 12-month-old C57BL/6 male mice (Harlan, Israel) were injected intraperitoneally (i.p.) with DEN (25 mg/g body wt; Sigma-Aldrich; St. Louis, MO). Mice were sacrificed at the indicated time points and liver tissues were fixed with 4% PFA or snap frozen in liquid nitrogen.

### Immunofluorescence

Livers were fixed in 4% formaldehyde, dehydrated, embedded in paraffin, and sectioned (5 μm-thick). To estimate DNA damage, sections were immuno-fluorescence stained with anti-phospho-histone H2AX (γH2AX) monoclonal antibody (Millipore, Billerica, MA) and rabbit anti-phospho-KAP-1 (s824; Bethyl Laboratories, Montgomery, TX), followed by the secondary antibodies Alexa-488 goat anti mouse IgG (Molecular Probes, Eugene, OR) and Alexa-647 donkey anti rabbit IgG (Jackson ImmunoResearch,West Grove, PA). Nuclei were counterstained with DAPI (Calbiochem, Darmstadt, Germany). An Olympus BX61 microscope (Olympus, Tokyo, Japan) was used for low field image acquisition and a laser scanning confocal microscope system (FluoView-1000; Olympus) with a 40X UPLAN-SApo objective and 2x digital zoo for high field acquisition. Positive γH2AX areas in mice livers were calculated using ImageJ software.

### RNA extraction

Total RNA was isolated from liver samples using Trizol reagent (Life Technologies, Paisley, UK) and purified using MaXtract High Density (QIAGEN, Redwood City, CA). RNA yield and quantity was determined using a Nanodrop spectrophotometer ND-1000 (Thermo scientific, Wilmington, DE). The RNA quality was tested using a ND-1000 V3.7.1, according to the manufacturer's instructions and assigned an RNA integrity number (RIN). Only samples with a RNA integrity number (RIN) of 8 or greater were employed.

### RNAseq and bioinformatic analysis

Each sample represents equal amount of total RNA from three mice that were pooled prior to library construction. RNAseq libraries were constructed in the center for Genomic Technologies using Trueseq RNA Library preparation kit according to Illumina protocol and sequenced with the Illumina Nextseq 500 System to an average depth of around 30 million reads. The bioinformatics' analyses were performed in the Bioinformatics Unit of the I-CORE Computation Center at the Hebrew University and Hadassah. The NextSeq base-calls files were converted to fastq files using the bcl2fastq (v2.17.1.14) program. The processed fastq files were mapped to the mouse transcriptome and genome using TopHat (v2.0.13). The genome version was GRCm38, with annotations from Ensembl release 84. Normalization and differential expression were performed with the DESeq2 package (version 1.10.1). Differential expression was calculated using a design, which included the age factor, the days post-treatment factor and the interaction between them, compared with a reduced model that lacked the interaction term, and using the LRT test (all other parameters were kept at their defaults). The significance threshold for all comparisons was taken as the padj<0.1. Results were then combined with gene details (such as symbol, Entrez accession, etc.) taken from the results of a BioMart query (Ensembl, release 84) to generate a final Excel file. The ClueGO application (http://www.cytoscape.org) was applied to gene lists that were significantly differentially expressed. ClueGO visualizes the non-redundant biological terms for large clusters of genes in a functionally grouped network and performs network visualization of biological function. Gene ontology of biological process (July 2016) was used. Analysis for enriched pathways, upstream regulators and networks were also performed using QIAGEN's Ingenuity® Pathway Analysis (IPA®, QIAGEN, http://www.qiagen.com/ingenuity). In addition a protein interaction network was constructed for the differentially expressed genes of the various comparisons. Since protein-protein interaction data is about 10-fold larger for human than for mouse (based on BioGRID statistics, http://wiki.thebiogrid.org/doku.php/statistics), we built the network using the human orthologs. Protein interaction data was extracted from the Human Integrated Protein-Protein Interaction Reference (HIPPIE 2.0, June 2016; [40]). The network was visualized using Cytoscape.

### Validation of RNAseq Results by quantitative Real-time PCR (qRT-PCR)

To validate the results of RNAseq analysis, we confirmed by qRT-PCR the differential expression of some representative genes. Total RNA was reverse transcribed to cDNA using oligo(dT) primers and M-MLV reverse transcriptase (Thermo scientific). We used PerfeCTa SYBR Green FastMix ROX (Quanta Biosiences) for real-time PCR according to the manufacturer's protocol and all the samples were run in triplicate on CFX384 Touch Real-Time system c1000 thermal cycler (Bio-Rad, Hercules, CA). Cycling conditions were 95°C for 20 sec, followed by 40 cycles of 95°C for 1 sec, and 60°C for 20sec, 65°C for 5sec. Gene expression levels were normalized to Hprt gene. Primers used for qPCR analysis can be found in Supplementary table 1.

### Statistical analysis

All data were subjected to statistical analysis using the Excel software package (Microsoft) or GraphPad Prism6 (GraphPad Software Inc., La Jolla, CA, USA). Two-tailed Student's t- test was used to determine the difference between the groups. Data are given as mean ± SD or ±SEM, and are shown as error bars for all experiments. Differences were considered significant at P < 0.05.

## SUPPLEMENTARY MATERIAL FIGURES AND TABLE


