# Correction: Early age decline in DNA repair capacity in the liver: in depth profile of differential gene expression

**DOI:** 10.18632/aging.101242

**Published:** 2017-05-30

**Authors:** Avital Guedj, Anat Geiger-Maor, Hadar Benyamini, Yaval Nevo, Sharona Elgavish, Eithan Galun, Hagai Amsalem, Jacob Rachmilewitz

Original article: Aging (Albany NY) 2017; 8(11): 3131-3146.

PMCID: PMC 5191890 PMID: 27922819 doi: 10.18632/aging.101120

Please check the link to the original paper: http://www.aging-us.com/article/101120/text

**Present:** Due to an omission of three authors, the list of authors is incomplete.

**Corrected:** Additional authors - Hadar Benyamini, Yaval Nevo and Sharona Elgavish - are listed below. The authors sincerely apologize for this error.

**PRESENT LIST:**

**Avital Guedj^1^, Anat Geiger-Maor^1^, Eithan Galun^1^, Hagai Amsalem^2^, Jacob Rachmilewitz^1^**

^1^Goldyne Savad Institute of Gene Therapy, Hadassah-Hebrew University Medical Center, Jerusalem, Israel

^2^Department of Obstetrics and Gynecology, Hadassah University Hospital-Mount Scopus, Jerusalem, Israel

**UPDATED LIST:**

**Avital Guedj^1^, Anat Geiger-Maor^1^, Hadar Benyamini^2^, Yaval Nevo^2^, Sharona Elgavish^2^, Eithan Galun^1^, Hagai Amsalem^3^, Jacob Rachmilewitz^1^**

^1^Goldyne Savad Institute of Gene Therapy, Hadassah-Hebrew University Medical Center, Jerusalem, Israel

^2^Bioinformatics Unit, of the I-CORE Computation Center, the Hebrew University and Hadassah Hebrew University Medical Center, Jerusalem, Israel

^3^Department of Obstetrics and Gynecology, Hadassah University Hospital-Mount Scopus, Jerusalem, Israel

