# Supplementary material for: Early age decline in DNA repair capacity in the liver: in depth profile of differential gene expression
Source: Aging (Albany NY). 2016 Nov 30;8(11):3131–46. doi: 10.18632/aging.101120 (PMC5191890; doi:10.18632/aging.101120)
Supplement: Supplementary file 1 [file aging-08-3131-s001.pdf]

SUPPLEMENTARY MATERIAL

A

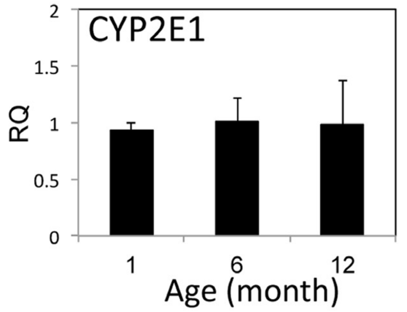

B

General_Gene_Details		Normalized_Counts_per_Sample				C_6M / C_3W					Significance
Ensembl_Gene_ID	Associated_Gene_Name	C_3W_1	C_3W_2	C_6M_1	C_6M_2	og2FoldChang	lfcSE	stat	pvalue	padj	C_6M / C_3W
ENSMUSG00000060407	Cyp2a12	17486.641	20720.850	11402.358	13498.510	-0.617	0.135	-4.560	5.107E-06	6.159E-04	yes
ENSMUSG00000091867	Cyp2a22	1038.661	1209.649	33.972	35.589	-5.012	0.428	-11.709	1.149E-31	2.632E-28	yes
ENSMUSG00000074254	Cyp2a4	8867.257	1104.807	12.133	12.604	-8.654	1.239	-6.984	2.875E-12	1.398E-09	yes
ENSMUSG00000030483	Cyp2b10	539.903	1124.189	211.110	40.037	-2.734	0.813	-3.362	7.750E-04	2.988E-02	yes
ENSMUSG00000040660	Cyp2b9	2004.064	1766.457	4.853	2.966	-8.952	0.949	-9.429	4.148E-21	5.118E-18	yes
ENSMUSG00000025004	Cyp2c40	4688.527	9038.446	129.820	166.822	-5.528	0.367	-15.072	2.493E-51	2.000E-47	yes
ENSMUSG00000062624	Cyp2c67	3124.012	4374.294	7160.749	8046.020	1.020	0.221	4.620	3.833E-06	4.879E-04	yes
ENSMUSG00000074882	Cyp2c68	9516.546	12652.414	3832.735	3773.889	-1.543	0.236	-6.526	6.769E-11	2.857E-08	yes
ENSMUSG00000092008	Cyp2c69	2722.597	4554.023	30.332	54.125	-6.408	0.446	-14.353	1.018E-46	4.084E-43	yes
ENSMUSG00000023963	Cyp39a1	2693.495	2759.373	463.471	368.492	-2.714	0.335	-8.114	4.916E-16	3.943E-13	yes
ENSMUSG00000054417	Cyp3a44	69.244	115.414	6.066	8.156	-3.676	0.677	-5.431	5.603E-08	1.362E-05	yes
ENSMUSG00000061292	Cyp3a59	2779.799	4266.809	707.339	1289.350	-1.818	0.329	-5.517	3.451E-08	8.516E-06	yes
ENSMUSG00000066071	Cyp4a12a	2491.784	5445.622	8244.203	8639.165	1.089	0.339	3.211	1.324E-03	4.240E-02	yes
ENSMUSG00000078597	Cyp4a12b	440.553	940.055	2920.353	2962.762	2.091	0.449	4.660	3.160E-06	4.087E-04	yes
ENSMUSG00000039519	Cyp7b1	663.338	1494.220	4651.696	9310.903	2.694	0.389	6.922	4.455E-12	2.042E-09	yes

C

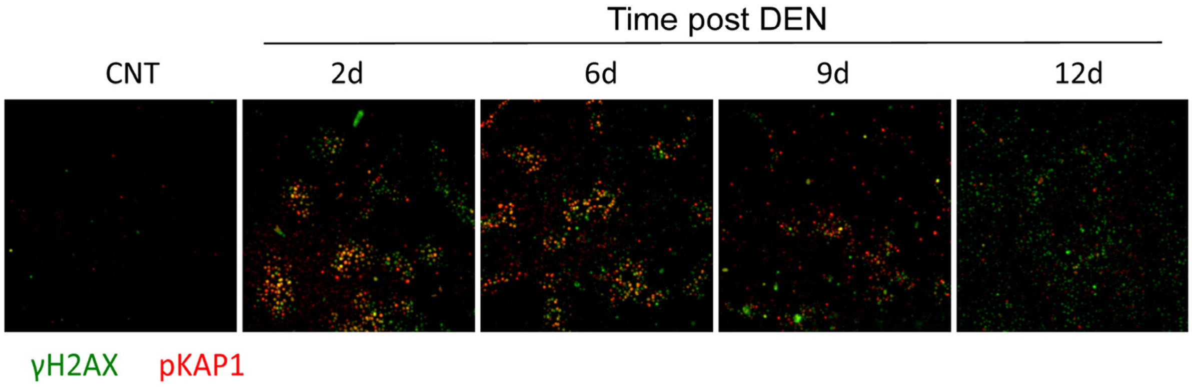

Supplementary Figure S1. Delayed DNA damage response in 6-month-old mice. (**A**) RT-qPCR analysis of CYP2E1 expression in RNA extracts from frozen liver tissues harvested from mice at the indicated ages. (**B**) A table presenting RNAseq data for the 15 CYP genes that show differential expression between 1 and 6 month-old mice. 11 genes were downregulated with age and only five genes were upregulated. (**C**) Six-month-old mice were injected with DEN and the levels of residual damage before and after 2, 6, 9 and 12 days were determined as above. Representative images of γH2AX (green) and pKAP-1 (red) staining are shown.

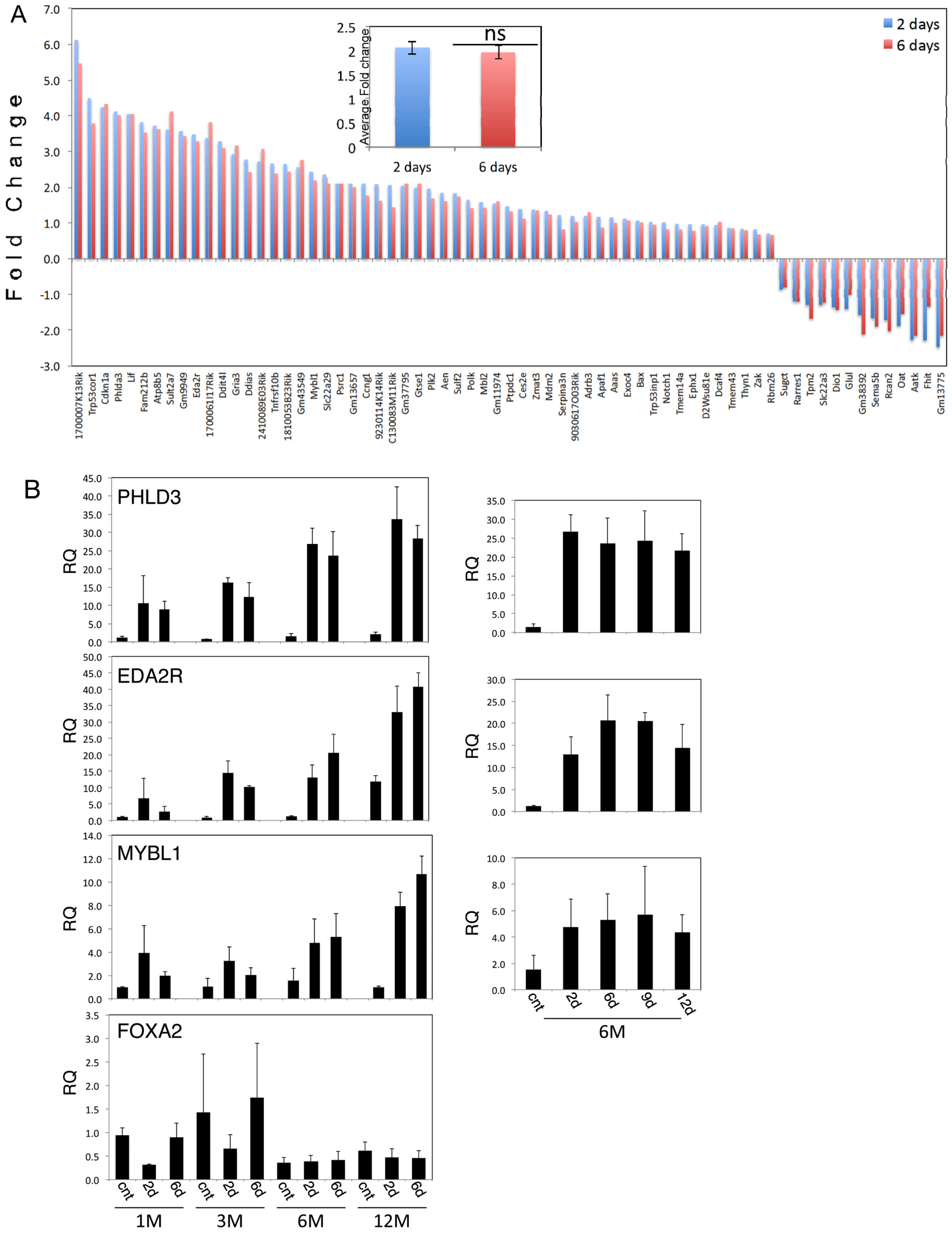

Supplementary Figure S2. Gene expression profiles following DEN treatment. (**A**) Plot depicting the fold-change of 67 transcripts that are differentially expressed both in 2 days (blue bars) and 6 days (red bars) after DEN treatment in 6-month-old mice. Inset shows the average fold changes of all 67 genes ±SD. ns. non-significant. (**B**) qRT-PCR analysis of genes representing differential gene expression patterns in response to DEN treatment. Left panels: The level of gene expression in 1, 3, 6 and 12-month old mice before and 2 and 6 days after DEN treatment. Right panels: the level of gene expression before and 2, 6, 9 and 12 days after DEN treatment in 6-month-old mice.

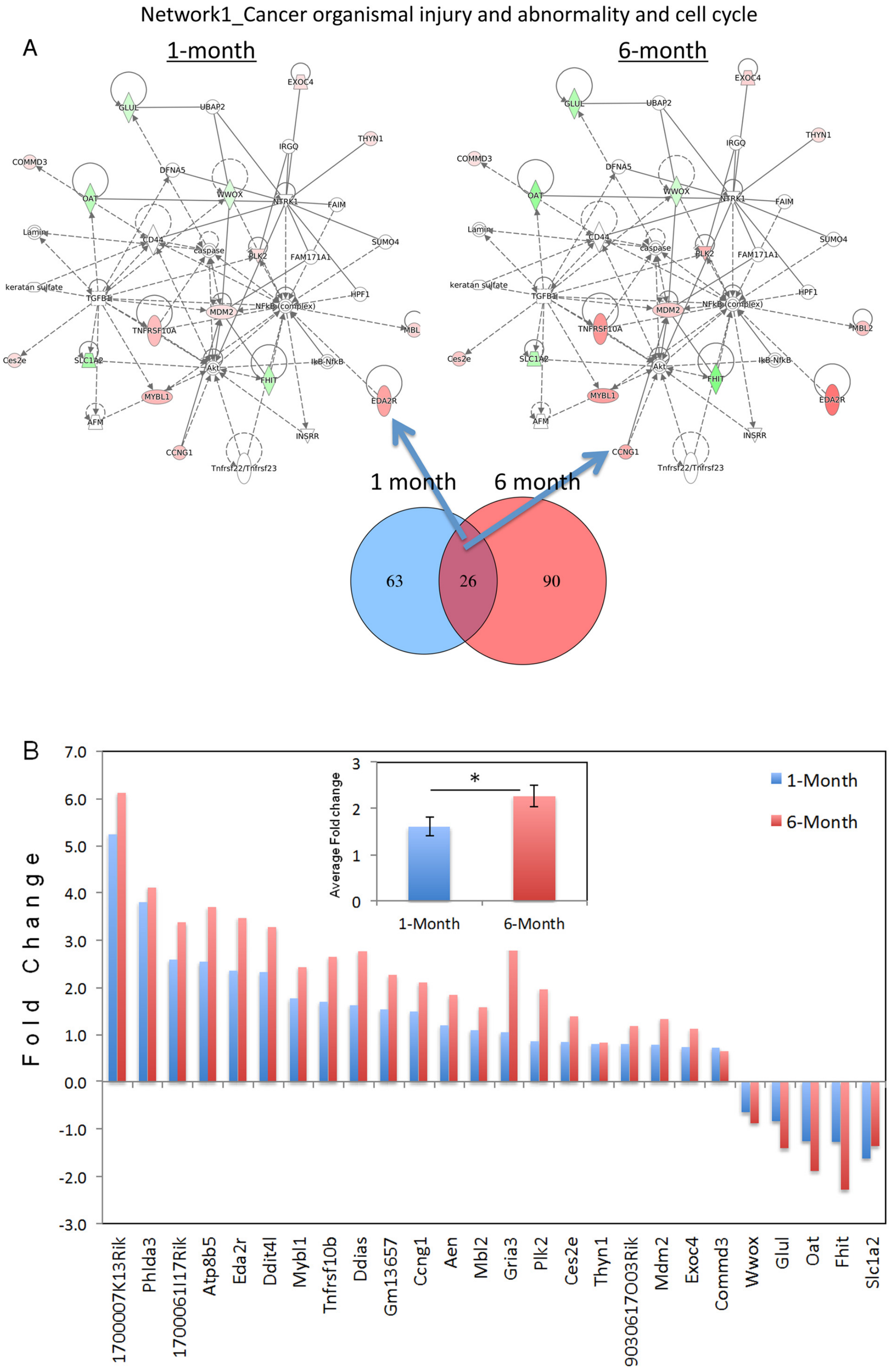

Supplementary Figure S3. DEN induce a robust transcriptional response in 6-month-old mice. (**A**) Age-related IPA enriched interaction network for 26 differentially expressed transcripts 2 days after DEN that are common for both 1- and 6-month-old mice. Each gene is colored according to the directionality of expression; red-upregulated, green-downregulated. Color intensity is roportional to the fold change of that particular gene. (**B**) Graph depicting the fold-change of all 26 transcripts that are differentially expressed in both 1-month (blue bars) and 6-month old mice (red bars) 2 days after DEN treatment. Inset shows the average fold changes of all genes for the two ages ±SD. * $p<0.05$.

A

Upstream Regulators (Activated)	Activation z-score	p-value of overlap	Target molecules in dataset
cisplatin	2.651	3.25E-05	CCNG1,ENDOG,FOS,GDF15,JUND,MDM2,OAT,PHLDA3,PLK2,ZNF174
IL1B	2.387	5.63E-03	DDIAS,FOS,G0S2,GDF15,IL1R1,JUND,SLC1A2,TNFRSF10A
hydrogen peroxide	2.328	2.93E-03	DDIAS,FOS,G0S2,GDF15,IL1R1,JUND,SLC1A2,TNFRSF10A
[illegible]	2.000	3.57E-04	[illegible]

B

Upstream Regulators (Activated)	Activation z-score	p-value of overlap	Target molecules in dataset
TP53	3.937	1.53E-20	ADRB3,AEN,APAF1,BAX,BCL2L1,BTG2,CCNG1,CDKN1A,CEP170B,DDIAS,DDIT4L,DUSP6,EDA2R,EPHX1,FAM212B,GAS6,GLUL,GNA14,GTSE1,LIF,MDM2,MYBL1,NOTCH1,OAT,PARK2,PHLDA3,PLK2,POLK,PSRC1,SERPINA3,SESN2,TMEM43,TNFRSF10A,TP53INP1,Tpm2,ZMAT3
TNF	3.623	4.20E-06	AATK,ADRB3,APAF1,BAX,BCL2L1,BTG2,CDKN1A,DDIAS,DIO1,DUSP6,LIF,MBL2,NFE2L2,NOTCH1,PLK2,RCAN2,SERPINA3,SLC1A2,TNFRSF10A,TP53INP1,ZAK
CDKN2A	3.239	3.08E-08	AK1,APAF1,BAX,BTG2,CCNG1,CDKN1A,GTSE1,MDM2,POLK,TP53INP1,ZMAT3
IL1B	2.639	5.97E-05	BAX,BCL2L1,BTG2,CDKN1A,CYP8B1,DDIAS,DIO1,GAS6,LIF,NFE2L2,SERPINA3,SLC1A2,TNFRSF10A
NFE2L2	2.597	4.62E-05	APOA4,BCL2L1,CDKN1A,Cyp2a12/Cyp2a22,EPHX1,GNA14,NFE2L2,OAT,SERPINA3
methyl methanesulfonate	2.596	5.47E-10	BTG2,CCNG1,CDKN1A,LIF,MDM2,PHLDA3,TP53INP1
doxorubicin	2.434	6.15E-07	APAF1,BAX,BCL2L1,BTG2,CDKN1A,DDIAS,MDM2,NFE2L2,PSRC1,TNFRSF10A,TP53INP1
1,2-dithiol-3-thione	2.433	2.56E-04	APOA4,Cyp2a12/Cyp2a22,EPHX1,GNA14,NFE2L2,SERPINA3
topotecan	2.415	1.28E-05	CDKN1A,FHIT,MDM2,PARD3B,PARK2,PLK2,SESN2
FGF2	2.401	6.56E-04	BAX,BCL2L1,BTG2,CDKN1A,GRIA3,MDM2,NOTCH1
benzo(a)pyrene	2.400	3.62E-04	BAX,CDKN1A,EPHX1,GNA14,MDM2,ZMAT3
CHEK2	2.400	3.75E-11	APAF1,BAX,BCL2L1,CCNG1,CDKN1A,MDM2
KRAS	2.236	1.72E-05	BCL2L1,CDKN1A,DUSP6,GLUL,MDM2,NFE2L2,SERPINA3,TNFRSF10A,Tpm2
idarubicin	2.236	1.34E-08	BAX,CDKN1A,MDM2,SESN2,TP53INP1
arsenite	2.236	8.37E-05	CDKN1A,FHIT,MDM2,NFE2L2,WWOX
NFkB (complex)	2.219	8.28E-06	BAX,BCL2L1,CDKN1A,DIO1,GAS6,LIF,MBL2,MDM2,MYBL1,NOTCH1,PLK2,SERPINA3
SLC29A1	2.200	1.34E-08	BAX,CCNG1,CDKN1A,MDM2,TP53INP1
gentamicin	2.196	3.85E-03	BAX,BTG2,CCNG1,DIO1,MDM2
paclitaxel	2.186	3.58E-06	BAX,BCL2L1,BTG2,CCNG1,CDKN1A,DUSP6,MDM2,TNFRSF10A,TP53INP1
cytarabine	2.183	8.82E-06	BAX,CDKN1A,MDM2,SESN2,TP53INP1

C

Upstream Regulators (Activated)	Activation z-score	p-value of overlap	Target molecules in dataset
TP53	3.838	6.55E-18	ADRB3,AEN,APAF1,BAX,CCNG1,CDKN1A,DDIAS,DDIT4L,EDA2R,EPHX1,FAM212B,GLUL,GSTM1,GTSE1,LIF,MDM2,MYBL1,NOTCH1,OAT,PHLDA3,PLK2,POLK,PSRC1,SERPINA3,TMEM43,TNFRSF10A,TP53INP1,Tpm2,ZMAT3
TNF	3.011	2.01E-07	AATK,ADRB3,APAF1,BAX,CDKN1A,CFD,DDIAS,DIO1,FAM198B,GPD1,LIF,MBL2,NOTCH1,PLK2,RCAN2,SERPINA3,TNFRSF10A,TP53INP1,ZAK
CDKN2A	2.922	1.52E-07	APAF1,BAX,CCNG1,CDKN1A,GTSE1,MDM2,POLK,TP53INP1,ZMAT3
lipopolysaccharide	2.645	8.99E-03	BAX,CDKN1A,CFD,GPD1,GTSE1,LIF,MBL2,NOTCH1,PLK2,SERPINA3,Tpm2
doxorubicin	2.497	1.68E-05	APAF1,BAX,CDKN1A,DDIAS,MDM2,PSRC1,TNFRSF10A,TP53INP1
IFNG	2.441	4.15E-03	BAX,CDKN1A,DDIAS,DIO1,GLUL,LIF,MDM2,NOTCH1,RARRES1,TNFRSF10A
methyl methanesulfonate ethanol	2.401	3.09E-09	CCNG1,CDKN1A,LIF,MDM2,PHLDA3,TP53INP1
benzo(a)pyrene	2.401	5.19E-05	BAX,CDKN1A,EPHX1,GSTM1,MDM2,ZMAT3
mitomycin C	2.374	2.70E-08	BAX,CDKN1A,MDM2,PSRC1,TP53INP1,ZMAT3
SLC29A1	2.219	2.25E-09	BAX,CCNG1,CDKN1A,MDM2,TP53INP1
ETS1	2.215	4.33E-04	BAX,CCNG1,CDKN1A,MDM2,MGAT5
NR1I2	2.207	7.00E-05	CDKN1A,GLUL,GSTM1,Gstm3,Mup1 (includes others)
CHEK2	2.186	8.53E-10	APAF1,BAX,CCNG1,CDKN1A,MDM2
doxifluridine	2.176	1.21E-09	BAX,CCNG1,CDKN1A,MDM2,TP53INP1
ethanol	2.156	4.26E-03	BAX,CDKN1A,EPHX1,Gstm3,TP53INP1
cisplatin	2.067	4.03E-08	BAX,CCNG1,CDKN1A,CLSTN3,GSTM1,MDM2,NOTCH1,OAT,PHLDA3,PLK2,PSRC1,TP53INP1,ZMAT3
ZBTB33	2.000	9.52E-08	APAF1,BAX,CDKN1A,TNFRSF10A
RASSF1	2.000	1.15E-04	CDKN1A,LIF,PLK2,TP53INP1
INHA	2.000	2.53E-04	BAX,EPHX1,TP53INP1,Tpm2
Idarubicin	2.000	2.68E-07	BAX,CDKN1A,MDM2,TP53INP1

Supplementary Figure S4. Upstream regulators and protein interaction network analysis. IPA upstream regulator analysis for differentially expressed genes 2 days after DEN treatment in 1-month (**A**) and 6-month-old mice (**B**; top 20 are shown) as well as 6 days after DEN in 6-month-old mice (**C**).

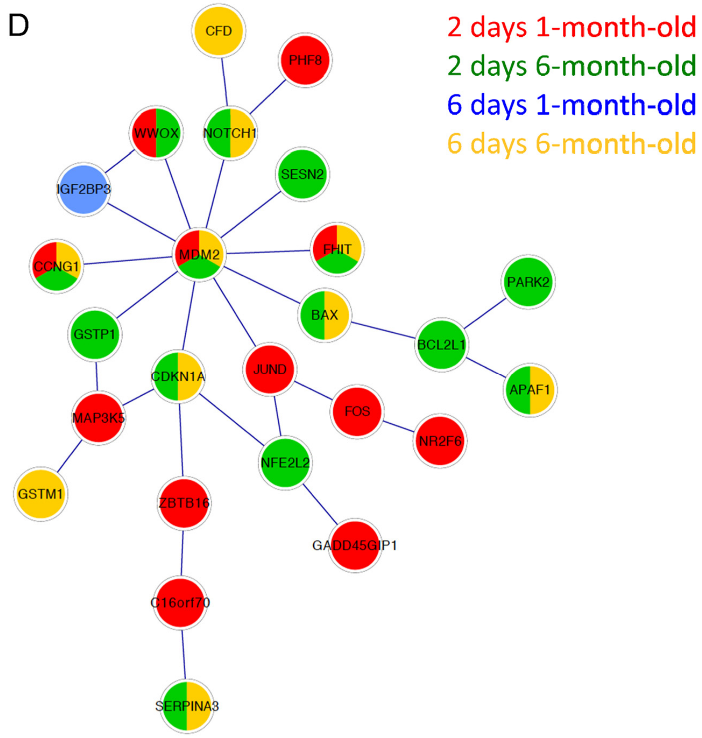

Supplementary Figure S4. Upstream regulators and protein interaction network analysis. (**D**) Protein:protein interaction network constructed for the differentially expressed genes of the various comparisons. Nodes representing interacting proteins and were colored as a pie chart based on their differential expression in any of the four indicated comparisons.

Supplementary Table 1. Oligonucleotide Sequences for qRT-PCR.

Target gene	Forward Primer	Reverse Primer
MYBL1	5'-GAAACCGTTGGGCCGAGATT-3'	5'-CCATCTTGTAAATAGCCCTCCTG-3'
Eda2r	5'-CACACTGCATAGTCTGCCCTC-3'	5'-GCCTTCTGGACCCGATTGA-3'
Foxa2	5'- CATGGGACCTCACCTGAGTC-3'	5'- CATCGAGTTCATGTTGGCGTA-3'
PHLDA3	5'-CCGTGGAGTGCGTAGAGAG-3'	5'-CCAGGGTGATCTGAGCGTT-3'
Cyp2e1	5'-CCACCAGCACAACTCTGAGATA-3'	5'-CCCAATAACCCTGTCAATTTCTT-3'
HPRT	5'-GCGATGATGAACCAGGTTATGA-3'	5'-ATCTCGAGCAAGTCTTTCAGTCCT-3'
